# Impairments to the multisensory integration brain regions during migraine chronification: correlation with the vestibular dysfunction

**DOI:** 10.3389/fnmol.2023.1153641

**Published:** 2023-07-03

**Authors:** Liang Dong, Xiaoping Fan, Yulan Fan, Ximao Li, Hui Li, Jiying Zhou

**Affiliations:** ^1^Department of Neurology, The First Affiliated Hospital of Chongqing Medical University, Chongqing, China; ^2^Department of Hospice, The First Affiliated Hospital of Chongqing Medical University, Chongqing, China; ^3^Department of Radiology, The First Affiliated Hospital of Chongqing Medical University, Chongqing, China

**Keywords:** migraine, chronification, vestibular dysfunction, functional connectivity, multisensory integration, resting-state functional magnetic resonance

## Abstract

**Objectives:**

Migraine is often combined with vestibular dysfunction, particularly in patients with chronic migraine (CM). However, the pathogenesis of migraine chronification leading to vestibular dysfunction is not fully understood. The current study investigated whether structural or functional impairments to the brain during migraine chronification could be associated with vestibular dysfunction development.

**Methods:**

The eligible participants underwent clinical assessment and magnetic resonance imaging (MRI) scans. Voxel-based morphometry (VBM) determined structural impairment by evaluating alterations in gray matter volume (GMV). Functional impairment was assessed by the mean amplitude of low-frequency fluctuation (mALFF). Furthermore, the resting-state functional connectivity (rsFC) of regions possessing impairment was examined with a seed-based approach. We also analyzed the correlations between altered neuroimaging features with clinical variables and performed multiple linear regression.

**Results:**

Eighteen CM patients, 18 episodic migraine (EM) patients, and 18 healthy controls (HCs) were included in this study. A one-way ANOVA indicated the group differences in mALFF. These were located within right supramarginal gyrus (SMG), left angular gyrus (AG), middle frontal gyrus (MFG), left middle occipital gyrus (MOG), right rolandic operculum (Rol) and left superior parietal gyrus (SPG). During rsFC analysis, the CM group had more enhanced rsFC of left SPG with left MOG than the EM and HC groups. The EM group revealed enhanced rsFC of left SPG with left AG than the CM and HC groups. In multiple linear regression, after controlling for age, body mass index (BMI) and disease duration, the rsFC of left SPG with left MOG (β = 48.896, *p* = 0.021) was found to predict the total Dizziness Handicap Inventory (DHI) score with an explained variance of 25.1%. Moreover, the rsFC of left SPG with left MOG (β = 1.253, *p* = 0.003) and right SMG (β = −1.571, *p* = 0.049) were significant predictors of migraine frequency, accounting for a total explained variance of 73.8%.

**Conclusion:**

The functional impairments due to migraine chronification are primarily concentrated in the multisensory integration-related brain regions. Additionally, the rsFC of SPG with MOG can predict the frequency of migraine and the degree of vestibular dysfunction. Therefore, these neuroimaging features could be potential mechanisms and therapeutic targets for developing vestibular dysfunction in migraine.

## Introduction

Migraine is a common primary headache disorder with a one-year worldwide prevalence estimated at 15% (GBD 2016 Neurology Collaborators, 2019). Migraine is highly disabling and contributes significantly to the global disease burden surpassing the burden of all other neurological disorders combined (Ashina et al., 2021). Recurrent headaches are characteristics of migraine, often accompanied by nausea, vomiting, photophobia, and phonophobia (Author, 2018).

As a sensory processing disorder, headache attacks are only the most typical symptom of migraine (Goadsby et al., 2017). Vestibular symptoms and balance disorders are more common in migraineurs than in the general population. The incidence of vertigo is 2–3 times higher in migraineurs than in those without headaches (Vuković et al., 2007). A cross-sectional study revealed that falls and balance impairment are more prevalent in migraine patients (54 and 69%) than in healthy controls (HCs) (2 and 2%) (Carvalho et al., 2018). The Barany Society has established diagnostic criteria for vestibular migraine (VM) due to the significant correlation between vestibular dysfunction and migraine. These are also included in the International Classification of Headache Disorders Appendix – third edition (ICHD-3) (Lempert et al., 2012; Author, 2018). However, the relationship between migraine and vestibular dysfunction is more complex. Vestibular dysfunction may be prevalent even in migraineurs without vestibular symptoms such as vertigo and dizziness (Bernetti et al., 2018; Zorzin et al., 2020; Carvalho et al., 2022), indicating that vestibular dysfunction may be inherent to migraine.

The link between migraine and vestibular dysfunction is unexplored. However, many studies have depicted a correlation between vestibular dysfunction and migraine chronification (Carvalho et al., 2017, 2019, 2022). Migraine chronification is the increasing frequency of headache attacks demonstrating the eventual progression from episodic migraine (EM) to chronic migraine (CM). Several studies by Carvalho et al. have observed that migraineurs with high headache frequency can have more vestibular symptoms (Carvalho et al., 2017, 2019, 2022; Zorzin et al., 2020). One of their studies found that 85% of female CM patients had vestibular symptoms. Thus, the increasing frequency of headaches can predict the severity of vestibular symptoms (Carvalho et al., 2019). Another recent clinical case-control study indicated vestibular symptom progression is associated with migraine chronification (Faim et al., 2022). Animal studies also prove that migraine chronification exacerbates the impairment of the central vestibular system pathways (Zhang et al., 2020; Tian et al., 2022). Therefore, this evidence suggests that a higher headache attack frequency further aggravates vestibular dysfunction.

Recent advancements in magnetic resonance imaging (MRI) technology allow non-invasive investigation of brain dysfunction and neurological disease. Many neuroimaging studies have identified structural and functional changes in brain regions associated with sensory processing in individuals with migraine (Schwedt et al., 2013; Hu et al., 2019; Dai et al., 2021; Lim et al., 2021; Zhang et al., 2023). These included vestibular information processing and those related to nociceptive processing. A recent meta-analysis found that migraineurs had gray matter volume (GMV) alterations across multiple brain regions associated with sensory, affective, and painful descending modulation (Zhang et al., 2023). A functional MRI study by Schwedt et al. (2013) observed significant differences in functional connectivity (FC) between the insula and other sensory processing regions in CM than in HCs. In addition, alterations in brain regions associated with sensory processing, such as the parietal and occipital lobes (Hu et al., 2019), somatosensory cortex (Lim et al., 2021), and cerebellum (Dai et al., 2021), have also been reported. Moreover, alterations in brain regions can aggravate the progression of migraine chronification (Lee M. et al., 2019; Magon et al., 2019; Chen et al., 2020). Studies have identified that these alterations can correlate with clinical parameters like examinations and scales (Woldeamanuel et al., 2019; Chen et al., 2020; Lai et al., 2020), reinforcing neuroimaging studies. However, it is unclear whether there is a correlation between the brain alterations due to migraine chronification and vestibular dysfunction development. These findings could have important implications for deepening the understanding of migraine chronification.

This study utilized voxel-based morphometry (VBM) (Ashburner and Friston, 2000) and mean amplitude of low-frequency fluctuation (mALFF) (Yang et al., 2007) for investigating brain GMV and spontaneous neural activity differences in EM, CM, and HCs. Regions with structural and functional alterations were used as region of interest (ROI) for resting-state functional connectivity (rsFC) analyses to explore neural activity consistency during migraine chronification. Meanwhile, structural questionnaires, vestibular examinations and related scales assessed headache characteristics, and vestibular function. Finally, correlation analyses between brain alterations and clinical variables aimed to reveal potential clinical implications. It was hypothesized that migraine chronification could lead to structural and functional impairment of the brain regions associated with sensory processing, leading to vestibular dysfunction.

## Materials and methods

### Participants

All the participants were recruited from the headache clinic of the First Affiliated Hospital of Chongqing Medical University between July 2021 and December 2022. They were diagnosed based on the ICHD-3 criteria of episodic or CM without aura. The age and gender ratio were matched across the three groups. Due to the unique pathophysiological mechanism of VM, patients who met the diagnosis of VM were excluded. The inclusion criteria were: (1) satisfying the diagnostic criteria in ICHD-3 1.1 for migraine without aura or 1.3 for CM; (2) 18–60 years of age; (3) migraine attack at least once a month within the last 3 months; (4) at least 6 months of migraine history; (5) not on any migraine preventive medication in the past 3 months; and (6) right-handed. Participants with the following conditions were excluded: (1) meet the diagnostic criteria for A.1.6.6 VM; (2) any other primary or secondary headache; (3) alcohol or substance abuse; (4) any other chronic pain disorder; (5) any vestibular disorder, including vestibular neuritis and Meniere’s disease; (6) any neurological, mental, or systemic disorders; (7) any contraindications to MRI scanning; and (8) pregnant or lactating. Right-handed HCs were recruited from the society and their age and sex were matched to the patients. The exclusion criteria for HCs were the same as those for patients from (2) to (8). In addition, any primary or secondary headache would be excluded. In this study, as we did in the exclusion criteria, we made efforts to exclude vestibular symptoms caused by non-migraine etiologies, including both central and peripheral causes. Primary vestibular symptoms in present study including vertigo, dizziness, vestibulovisual, and postural symptoms which were classified according to the Barany Society’s Classification of Vestibular Symptoms (Bisdorff et al., 2009). Eventually, 18 HCs, 18 patients with MwoA, and 18 CM patients were included in this study. All the MRI scans for migraineurs were conducted during the interictal period. On the morning of the scan day, participants received a series of vestibular tests, and later on the same day, participants underwent the MRI scan. Patients were asked to report whether they were awake and headache free during the scan. The study was done when patients were not having headache attack episodes, if the patient suffered a migraine attack or took acute medication within the 12 h before or after the scan, then the scan date would be rescheduled. All the participants were also required to abstain from consuming substances that may affect neural activity, such as alcohol and caffeine, on the day before and the day of the scan. The study was approved by the Ethics Committee of First Affiliated Hospital of Chongqing Medical University and all the participants provided informed consent.

### Clinical parameters

The demographic information such as age, gender, body mass index (BMI), and family history of the participants was recorded. A structured questionnaire collected headache characteristics of patients. The number of migraine days per month was utilized to assess the frequency of migraine attacks. A 10-point visual analog scale (VAS) helped rate headache intensity. Headache Impact Test (HIT-6) assessed the severity and impact of headaches. Headache-related disability was evaluated by the Migraine Disability Assessment Scale (MIDAS). The severity of vestibular dysfunction was assessed by Dizziness Handicap Inventory (DHI) (Jacobson and Newman, 1990). DHI was used to assess the impact of dizziness on individuals’ functional, emotional, and physical aspects through a 25-item questionnaire. Mini-Mental State Examination (MMSE) was used to assess general cognitive function. All the participants needed to have an MMSE score ≥ of 27. Anxiety and depression symptoms in patients were evaluated with the Hospital Anxiety and Depression Scale (HADS) (Mykletun et al., 2001).

### Vestibular function tests

#### Caloric test

The caloric test was performed using the Videonystagmography (VNG) and Air Caloric System (VertiGoggles ZT-VNG-II, Shanghai ZEHNIT Medical Technology Co., Ltd., Shanghai, China). It was conducted in the supine position with 30° angle head flexion and air irrigator airflow of 8 L/min at 46 and 24°C within 1 min, with a 5-min interval between irrigations. A binocular video-oculography system was used to measure horizontal eye movements during the study. After each exposure to the airflow, the maximum slow phase velocity (SPV) of nystagmus was determined. Directional preponderance (DP) and canal paresis (CP) were calculated depending on the SPV with the Jongkees method (Hallpike, 1975). The sum of the maximum nystagmus SPV for all four irrigations was recorded as the total response.

#### Video head impulse test

The Video Head Impulse Test (vHIT) is a diagnostic tool used to differentiate between various vestibular disorders. It assesses the gain of the vestibulo-ocular reflex (VOR) of each semicircular canal. VertiGoggles (ZT-VNG-II, Shanghai ZEHNIT Medical Technology Co., Ltd., Shanghai, China) was used to record VOR. The system utilizes lightweight goggles that have an integrated camera securely mounted to the head. Patients were asked to gaze at a dot on the television screen 1.2 m away while the technician manipulated the head of the patient with rapid and unpredictable head movements. During the tests, the targeted head velocities were 150–250°/s. The ratio of the mean eye velocity to the mean head velocity depicts the velocity gain of the VOR. The test was performed multiple times on each side, with at least 20 repetitions, to ensure stable results. The VOR gain for the right anterior canal (RA), left anterior canal (LA), the right lateral canal (RL), left lateral canal (LL), the right posterior canal (RP), and left posterior canal (LP) were recorded separately.

#### Oculomotor assessment

The oculomotor assessment was evaluated using VNG (VertiGoggles ZT-VNG-II, Shanghai ZEHNIT Medical Technology Co., Ltd., Shanghai, China). The saccade test was selected, allowing the results to be recorded as a continuous variable since this study needed correlational analysis. The participant was seated 1 m in front of a television screen. Participants were asked to keep their heads stationary while moving their eyes based on the instructions on the screen. During the saccade examination, participants were first asked to gaze at a small white dot at the center of the screen. Then, the target was moved 10° or 20° in pseudorandom directions at intervals greater than 1 s. The participant was asked to trace the dot. The saccadic latency and accuracy are critical indicators to evaluate the saccade function. The delay between the target appearance and the primary saccade onset was saccadic latency, and saccadic accuracy was defined as (saccadic amplitude/target amplitude) %.

#### Magnetic resonance imaging data acquisition

The MRIs were conducted with a 3.0-T MRI scanner (Ingenia, Philips Healthcare, Netherlands) and a 32-channel head coil. Functional images were acquired using an echo-planar imaging sequence with the following parameters: 48 slices, repetition time (TR) = 2,000 ms, echo time (TE) = 35 ms, 3.0-mm thickness, no gap, voxel size = 3.0 mm × 3.0 mm × 3.0 mm, flip angle (FA) = 90°, number of signal average (NSA) = 1, the field of view (FOV) = 230 mm × 230 mm, data matrix = 76 × 76, and 180 volumes. Moreover, the functional sequence took 6 min, and 6 s and structural images were obtained using a three-dimensional turbo spin echo T1WI sequence. It had the following parameters: 327 slices, TR/TE = 600/28.3 ms, 1.0-mm thickness, voxel size = 1.0 mm × 1.0 mm × 1.0 mm, FOV = 280 mm × 280 mm, an acquisition matrix = 280 × 280, NSA = 1, and FA = 90°. The structural sequence took 6 min and 5 s. Participants were asked to keep their eyes closed, remain still and stay awake during the entire scan.

### Data preprocessing

#### Structural data preprocessing

Structural features were quantified with VBM (Ashburner and Friston, 2000). T1 images were processed with Computational Anatomy Toolbox 12 (CAT12) toolbox for Statistical Parametric Mapping version 12 (SPM12) in MATLAB R2019a (MathWorks, Inc.) (Kurth et al., 2015). T1 images were preprocessed in CAT12 for each sample using recommended processing pipeline. It involved bias-field inhomogeneities correction, spatial registration, noise removal, skull stripping, gray matter, white matter, and cerebrospinal fluid (CSF) segmentation. All the T1 images were spatially normalized to the standard Montreal Neurological Institute (MNI) template with the DARTEL algorithm to a (1.5 mm × 1.5 mm × 1.5 mm) adult template. The resulting images were assessed for homogeneity. No images were discarded as they had a high correlation value (>0.85). Lastly, the structural data of each participant was smoothed with an 8-mm full width at half maximum (FWHM) Gaussian kernel. The further VBM analysis was conducted using the normalized and modulated GMV results after the above preprocessing.

#### Functional data preprocessing

The functional Image data preprocessing was operated using the DPABI version 6.2 software in MATLAB 2019a. The first 10 volumes were removed to avoid signal instability. Then, the fMRI images were slice timed and realigned for head motion. Any head motion data exceeding 1.5 mm or 1.5° in any direction were removed from the study. In the spatial normalization, the data were normalized to the MNI space, and the voxel size = 3 mm × 3 mm × 3 mm, using the DARTEL algorithm. The Friston 24 motion parameters, the white matter signal, and the cerebrospinal fluid (CSF) signal were regressed. After detrending, the data sets were bandpass filtered from 0.01 to 0.1 Hz. Finally, all the data were smoothed using a 4 mm FWHM Gaussian kernel (Yan et al., 2016).

#### Calculation of the mean amplitude of low-frequency fluctuation

The mALFF was determined using the DPABI software. Functional images without filtering were used to calculate mALFF. The time series of each voxel was converted into frequency domain data to generate the power spectrum using a fast-Fourier transform (FFT) technique. The ALFF value was determined as the average square root of the power spectrum from 0.01 to 0.08 Hz. The ALFF data for each voxel was divided by the global mean value to create a normalized value for further group comparison and standardization.

#### Calculation of seed-based functional connectivity

After VBM and mALFF analysis, abnormal regions were selected as seeds to explore ROI-based alterations in FC with other brain regions. The average time course for each seed was extracted for correlation analysis with the rest of the brain regions. Fisher’s Z-transformation normalized the resulting correlation coefficients (*r*) into standardized *Z* values which are more normally distributed and thus provide greater stability and accuracy in subsequent statistical analyses.

### Statistical analysis

#### Demographic and clinical data

The Statistical Package for the Social Sciences (SPSS) software package (version 26.0; SPSS Inc., Chicago, IL, United States) was utilized for statistical analysis. Demographic and clinical data among the three groups were evaluated using one-way ANOVA, *post hoc* analysis for the continuous variables, and Chi-squared tests for the categorical variables. A two-sample *t*-test was used to compare headache characteristics and vestibular function tests between the EM and CM groups. A *p* < 0.05 was considered the threshold for a statistically significant difference.

#### Structural and functional imaging features statistical analysis

Voxel-wise one-way ANOVA was conducted to investigate the group differences in mALFF values and rsFC with the DPABI software. Comparisons between groups for VBM data were performed using SPM12. The significance threshold was set at a *p* < 0.05 having false discovery rate (FDR) correction. At least a 20-voxel extension was required for the VBM and mALFF analyses and over 10 for rsFC analyses. Age and gender were considered covariates. Furthermore, *post hoc* analysis was performed to determine differences among each pair of the three groups. The group differences observed in the ANOVA were chosen as ROIs. Then, *post-hoc* tests were conducted in the ROIs with Bonferroni correction.

#### Correlation analysis between neuroimaging features and clinical variables

We extracted the average *Z*-values for each ROI and rsFC with significant differences to investigate the correlation between the brain region alteration and the clinical variables. We also performed Pearson correlation analysis with the clinical variables and neuroimaging features of patients using SPSS 22.0. A *p* < 0.05 was considered the threshold for a statistically significant difference. Then, we conducted a multiple linear regression on the obtained data to test whether neuroimaging features explained the degree of vestibular dysfunction (DHI score) and migraine frequency after adjusting for potential confounding factors. Migraine frequency and DHI score were used as dependent variables in separate models. We inputted our variables to the model in two steps starting with confounding factors, followed by neuroimaging features. We performed *post-hoc* tests to check for model assumptions, such as homoscedasticity (Residual scatter plots), multicollinearity (Variance Inflation Factor over 5) and the normal distribution (Histogram of residuals) of residuals. In addition, we assessed autocorrelation using the Durbin-Watson (D-W) value. When the D-W statistic is around 2, it indicates the absence of first-order autocorrelation in the model. In multiple regression analysis, it is advisable to limit the number of independent variables to 10–20% of the sample size for better model stability and to avoid overfitting. Given the smaller sample size in this study, we chose a 20% threshold for variable inclusion.

## Results

### Demographic and clinical characteristics

Eighteen eligible participants from each group were included in the study. The demographic and clinical variables of all the participants are represented in Table 1. No significant differences were observed in the three groups concerning age, gender, and BMI. Additionally, the two migraine subgroups showed no significant differences in the disease duration, headache intensity, HIT-6 score, and HADS scores. However, the CM and EM groups observed significant differences in MIDAS, migraine frequency and DHI.

**TABLE 1:** Demographic and clinical variables of all participants.

	CM (*n* = 18)	EM (*n* = 18)	HC (*n* = 18)	*F*(*t*)/χ ^2^	*p*-Value
Age (years)	38.83 ± 11.31	38.39 ± 8.88	38.72 ± 11.81	0.008	0.992
Gender (male/female)	3/15	3/15	3/15	0.000	1.000
BMI (kg/m^2^)	22.29 ± 2.71	21.39 ± 2.90	22.31 ± 3.08	0.595	0.556
Presence of vestibular symptoms	17/18 (94%)	13/18 (72%)		3.200	0.074
**Headache characteristics**					
Disease duration (years)	14.83 ± 9.24	10.89 ± 6.94		1.448	0.157
Frequency (days/month)	17.33 ± 7.31	3.50 ± 1.89		7.776	<0.001**
Intensity (0–10)	7.56 ± 0.98	7.78 ± 0.88		−0.715	0.479
HIT-6	64.72 ± 7.43	60.33 ± 7.38		1.777	0.084
MIDAS	64.00 ± 53.25	9.11 ± 7.38		4.332	<0.001**
**HADS**					
Anxiety score	4.22 ± 3.89	3.72 ± 2.91		0.437	0.665
Depression score	4.50 ± 4.18	3.44 ± 2.83		0.887	0.381
HADS-total score	8.72 ± 7.68	7.17 ± 5.49		0.699	0.489
**DHI**					
DHI-physical aspects	9.65 ± 8.70	3.54 ± 2.03		2.799	0.012*
DHI-emotional aspects	15.88 ± 11.63	8.31 ± 10.36		1.852	0.075
DHI-functional aspects	11.76 ± 8.91	6.62 ± 6.29		1.770	0.088
DHI-total score	37.29 ± 24.36	18.46 ± 16.68		2.388	0.024*

CM, chronic migraine; EM, episodic migraine; HC, healthy control; BMI, body mass index; HIT, Headache Impact Test; MIDAS, Migraine Disability Assessment Scale; HADS, Hospital Anxiety and Depression Scale; DHI, Dizziness Handicap Inventory. *p < 0.05; **p < 0.01.

### Vestibular function tests

All the CM, EM and HC participants completed caloric testing and saccade test. One CM patient could not cooperate in completing the vHIT test. Vestibular function tests of all the participants are depicted in Table 2. No statistical differences were observed in the CP and DP of the caloric testing. The results for vHIT were also not statistically different. In the saccade test, the three groups observed no significant difference in latency. ANOVA results showed differences between the three groups in terms of saccade accuracy, and *post-hoc* tests further revealed that the CM group was significantly less accurate than the EM (*p* = 0.0069; Bonferroni correction) and HC groups (*p* = 0.0044; Bonferroni correction), but there were no significant differences between the EM and HC groups.

**TABLE 2:** Results of vestibular function tests between the CM and EM groups.

	CM	EM	HC	*F* value	*p*-Value
**Caloric test**					
CP (%)	15.11 ± 9.57	11.50 ± 9.33	7.73 ± 4.78	2.558	0.089
DP (%)	11.61 ± 7.60	13.78 ± 7.75	10.18 ± 8.84	0.754	0.476
Total response (°/s)	78.21 ± 41.66	75.47 ± 31.80	85.74 ± 46.15	0.238	0.789
**Video head impulse test**					
RA VOR gain	1.02 ± 0.19	1.00 ± 0.15	1.04 ± 0.10	0.202	0.818
LA VOR gain	0.77 ± 0.17	0.84 ± 0.11	0.88 ± 0.06	2.939	0.063
RL VOR gain	1.14 ± 0.08	1.24 ± 0.19	1.13 ± 0.08	3.150	0.052
LL VOR gain	1.17 ± 0.08	1.27 ± 0.19	1.18 ± 0.08	2.686	0.079
RP VOR gain	0.94 ± 0.17	0.97 ± 0.15	1.01 ± 0.12	0.966	0.389
LP VOR gain	1.17 ± 0.25	1.11 ± 0.25	1.17 ± 0.08	0.600	0.553
**Saccade test**					
Saccade latency (ms)	200.78 ± 23.48	195.67 ± 23.48	201.29 ± 32.77	0.237	0.790
Saccade accuracy (%)	92.83 ± 7.64^†,^**^#^**	99.17 ± 5.53	99.93 ± 3.00	7.448	0.002**

CP, canal paresis; DP, directional preponderance; RA, right anterior canal; LA, left anterior canal; RL, right lateral canal; LL, left lateral canal; RP, right posterior canal; LP, left posterior canal; VOR, vestibulo-ocular reflex. ^†^*p* < 0.01 vs. HC; ^#^*p* < 0.01 vs. EM; *p < 0.05; **p < 0.01 ANOVA test.

### VBM and mALFF analysis

No statistically significant structural variations were identified among the three groups in the VBM analysis. A voxel-wise one-way ANOVA indicated that the intergroup differences in mALFF were located within the right supramarginal gyrus (SMG), the left angular gyrus (AG), left middle frontal gyrus (MFG), left middle occipital gyrus (MOG), right rolandic operculum (Rol), and the left superior parietal gyrus (SPG) (*p* < 0.05; FDR-corrected) (Table 3). *Post hoc* analysis revealed additional details (Table 4). The EM group had the highest mALFF values in the left AG, left MFG, and left MOG. In contrast, the HC group had the lowest values. Moreover, the CM group exhibited mALFF values intermediate between the EM and HC groups, showing statistical differences between all three groups within these regions (*p* < 0.05; Bonferroni correction). EM had the highest mALFF values in the SMG region, followed by the HC group. In contrast, the CM group had the lowest values, showing statistical differences between all three groups (*p* < 0.05; Bonferroni correction). The alterations of mALFF values in the right Rol were observed when comparing the CM and HC groups, and the values in the CM group were significantly higher than those in the HC group (*p* < 0.05; Bonferroni correction). The CM and EM groups in the left SPG showed significantly decreased mALFF values than the HC group. No statistical difference was observed between the CM and EM groups (*p* < 0.05; Bonferroni correction).

**TABLE 3:** Brain regions showing significant differences in mALFF among the HC, EM, and CM groups.

No.	Brain region	MNI coordinate			Voxels	*F* score
		*X*	*Y*	*Z*		
ROI 1	SupraMarginal_R	45	−45	36	116	33.9299
ROI 2	Angular_L	−45	−57	36	54	35.6429
ROI 3	Frontal_Mid_L	−24	33	30	40	24.6373
ROI 4	Occipital_Mid_L	−30	−69	33	27	25.8862
ROI 5	Rolandic_Oper_R	57	36	6	20	15.8235
ROI 6	Parietal_Sup_L	−21	−60	60	20	16.3331

The intergroup differences among the three groups were tested using one-way ANCOVA with age and gender as covariates. A threshold of *p* < 0.05 (FDR corrected) with a 20-voxel extension threshold was considered statistically different. ALFF, amplitude of low-frequency fluctuations; MNI, Montreal Neurological Institute; L, left; R, right; Mid, mid; Sup, superior; Oper, operculum; ROI, region of interest.

**TABLE 4:** *Post-hoc* two-sample *t*-test between CM and EM group.

Mask region	Peak coordinate region	MNI coordinate			Voxels	*T* score
		*X*	*Y*	*Z*		
**SupraMarginal_R**						
CM < EM	Parietal_Inf_R	48	−48	39	90	−5.7019
CM < HC	Angular_R	45	−57	33	62	−5.3696
EM > HC	SupraMarginal_R	45	−45	36	15	6.0031
**Angular_L**						
CM < EM	Angular_L	−45	−57	36	50	−6.0735
CM > HC	Occipital_Mid_L	−36	−63	30	19	5.4214
EM > HC	Angular_L	−45	57	36	29	5.5572
**Frontal_Mid_L**						
CM < EM	Frontal_Mid_L	−24	33	30	22	−4.7753
CM > HC	Frontal_Mid_L	−30	42	15	14	4.7286
EM > HC	Frontal_Mid_L	−24	33	30	25	5.2865
**Occipital_Mid_L**						
CM < EM	Occipital_Mid_L	−30	−69	33	18	−4.3287
CM > HC	Occipital_Mid_L	−33	−72	36	14	3.9597
EM > HC	Occipital_Mid_L	−30	−69	33	26	5.6096
**Rolandic_Oper_R**						
CM > HC	Frontal_Inf_Oper_R	48	6	18	19	4.2754
**Parietal_Sup_L**						
CM < HC	Parietal_Sup_L	−15	−63	54	17	−4.0791
EM < HC	Parietal_Sup_L	−21	−60	60	15	−4.3441

CM, chronic migraine; EM, episodic migraine; HC, healthy control; MNI, Montreal Neurological Institute; L, left; R, right; Mid, mid; Sup, superior; Oper, operculum.

### Functional connectivity analysis

**FIGURE 1:**
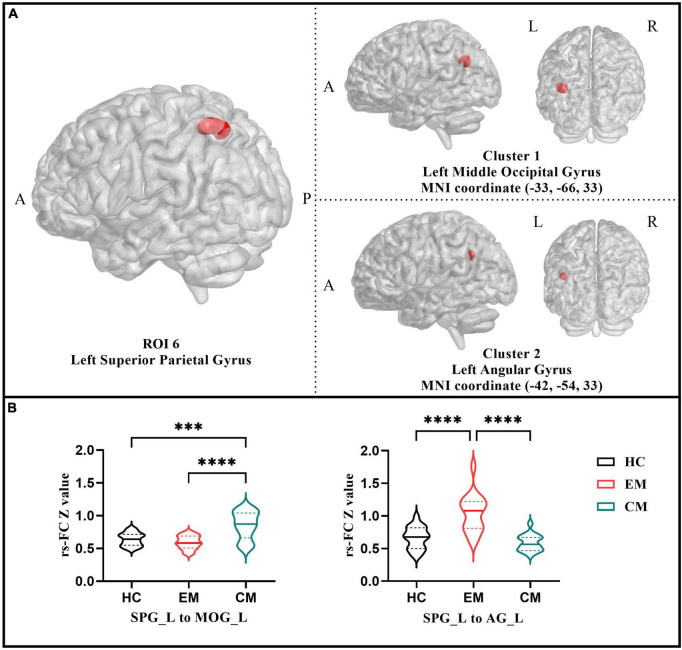
**(A)** Resting-state functional connectivity analysis results of the left superior parietal gyrus among three groups (*p* < 0.05 with FDR-corrected). **(B)** The violin plot displayed results of the *post-hoc* tests in the two functional connectivity based on left superior parietal gyrus (*p* < 0.05 with Bonferroni correction). CM, chronic migraine; EM, episodic migraine; HC, healthy control. ***p < 0.001; ****p < 0.0001.

The six ROIs were defined based on the regions showing significant differences in structural or functional imaging features in the current study (Table 3). RsFC differences between the left SPG to the left MOG and left AG were subsequently identified using one-way ANOVA analysis within the three groups (*p* < 0.05; FDR-corrected) (Figure 1A and Table 5). Specifically, the CM group revealed increased rsFC between the left SPG to the left MOG than both the EM (*p* < 0.0001; Bonferroni correction) and HC (*p* = 0.0002; Bonferroni correction) groups. The EM group indicated enhanced rsFC between the left SPG to the left AG compared to both the CM (*p* < 0.0001; Bonferroni correction) and the HC groups (*p* < 0.0001; Bonferroni correction) (Figure 1B).

**TABLE 5:** Regions showing rsFC differences based on the ROI 6 among all groups.

No.	Brain region	MNI coordinate			Voxels	*T* score
		*X*	*Y*	*Z*		
**Seed: Parietal_Sup_L**						
Cluster 1	Occipital_Mid_L	−33	−66	33	12	26.0232
Cluster 2	Angular_L	−42	−54	33	15	22.3581

MNI, Montreal Neurological Institute; L, left; R, right; Mid, mid; Sup, superior; rsFC, resting-state functional connectivity; ROI, region of interest.

### Correlation analyses

**FIGURE 2:**
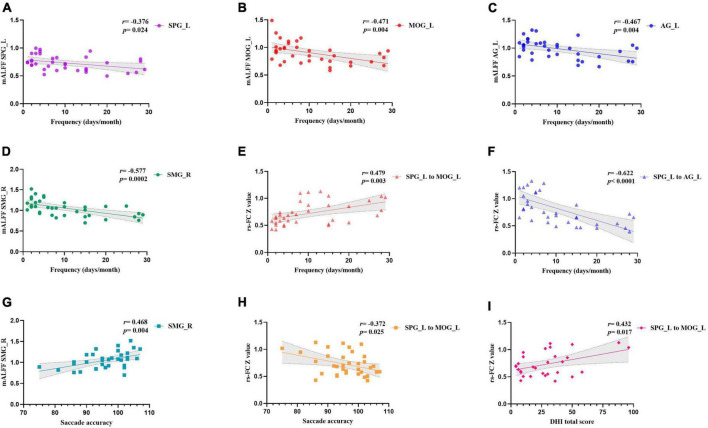
Correlation analysis of clinical profiles and the altered brain features. Panels **(A–D)** showed the correlation between headache frequency and mALFF values in different brain regions. Panels **(E,F)** showed the correlation between headache frequency and functional connectivity between left SPG with left MOG and left AG. Panel **(G)** showed the correlation between saccade accuracy and mALFF values in right SMG. Panel **(H)** showed the correlation between saccade accuracy and functional connectivity between left SPG with left MOG. Panel **(I)** showed the correlation between DHI total score and functional connectivity between left SPG with left MOG. SMG, supramarginal gyrus; AG, angular gyrus; MOG, middle occipital gyrus; SPG, superior parietal gyrus; L, left; R, right.

The migraine frequency showed a significant positive correlation with the rsFC between the left SPG to left MOG (*r* = 0.479, *p* = 0.003). Moreover, it had a significant negative correlation with left SPG (*r* = −0.376, *p* = 0.024), left MOG (*r* = −0.471, *p* = 0.004), left AG (*r* = −0.467, *p* = 0.004), right SMG (*r* = 0.479, *p* = 0.003), and the rsFC within the left SPG to left AG (*r* = −0.622, *p* < 0.0001). The saccade accuracy showed a significant negative correlation with rsFC between left SPG to left MOG (*r* = −0.372, *p* = 0.025) and a positive correlation with right SMG (*r* = 0.468, *p* = 0.004). The DHI total score was positively correlated with the rsFC between the left SPG and left MOG (*r* = 0.432, *p* = 0.017) (Figure 2).

Multiple linear regression was used to test whether neuroimaging features explained the degree of vestibular dysfunction in migraineurs, after controlling for age, BMI, and disease duration (Table 6). First, we set the total DHI score as the dependent variable. Then, we entered our variables to the model stepwise starting with age, BMI, and disease duration, followed by the mALFF value of right SMG (ROI 1) in the second, the *Z* value of the rsFC between left SPG to left MOG (ROI 6 to C1) in the third step which are the features obtained in correlation analysis that may be associated with vestibular dysfunction. We found that the ROI 6 to C1 was a significant predictor of total DHI score, where a higher *Z* value of ROI 6 to C1 (β = 48.896, *p* = 0.021) predicted a higher total DHI score, after controlling for age, BMI and disease duration. The total explained variance of the regression model was 25.1% (*R*^2^ = 0.251). The normal distribution of the standardized residual, homoscedasticity and multicollinearity were all fulfilled.

**TABLE 6:** Multiple linear regression model with neuroimaging features explaining DHI total score, after controlling for age, BMI, and disease duration.

Model	Predictors	β	SE	*T* score	*p*-Value	VIF	*R* ^2^	D-W value
1	Age	−0.402	0.595	−0.676	0.505	1.707	0.059	1.759
	BMI	1.783	1.997	0.893	0.380	1.503		
	Duration	0.456	0.608	0.749	0.461	1.387		
2	Age	−0.406	0.608	−0.668	0.510	1.712	0.059	1.755
	BMI	1.764	2.044	0.863	0.397	1.515		
	Duration	0.483	0.672	0.719	0.479	1.629		
	ROI 1	2.676	25.140	0.106	0.916	1.189		
3	Age	−0.132	0.565	−0.233	0.818	1.781	0.251	2.249
	BMI	1.296	1.872	0.692	0.495	1.531		
	Duration	0.525	0.612	0.857	0.400	1.630		
	ROI 1	16.456	23.566	0.698	0.492	1.259		
	ROI 6 to C1	48.896	19.745	2.476	0.021*	1.105		

*p < 0.05; n = 30. DHI, Dizziness Handicap Inventory; β, parameter estimate; SE, standard error; VIF, Variance Inflation Factor; D-W value, Durbin-Watson value; BMI, body mass index; ROI 6 to C1, the rsFC of left SPG to left MOG; ROI 1, right supramarginal gyrus.

Another multiple linear regression was preformed to test whether neuroimaging features can also explain migraine frequency. We set the migraine frequency as the dependent variable. Then, we inputted confounding factors. In the second step, we included the value of ROI 6 to C1 and ROI 1 because both of them demonstrated significant correlations with headache frequency and vestibular dysfunction indicators in the correlation analysis. In the third step, we further included the values of the *Z* value of the rsFC between the left SPG to left AG (ROI 6 to C2), the mALFF value of left MOG (ROI 4), and left SPG (ROI 6), as they showed significant correlations with headache frequency. ROI 2 was excluded from the regression analysis due to significant collinearity with ROI 6 to C2. However, during the model validation process, we discovered heteroscedasticity in the residuals, indicating model instability (Supplementary Table 1 and Supplementary Figures 1–3). To address this issue, we applied a logarithmic transformation with a base of 10 to all variables included in the model. We then followed the above steps to build the model again (Table 7). We found that the ROI 6 to C1 (β = 1.253, *p* = 0.003) and ROI 1 (β = −1.571, *p* = 0.049) was a significant predictor of migraine frequency. The total explained variance of the regression model was 73.8% (*R*^2^ = 0.738). The standardized residuals followed a normal distribution, homoscedasticity was satisfied, and there were no issues of multicollinearity (Supplementary Figures 4, 5). Despite the recommended limit of 7 independent variables based on a sample size of 36, we included 8 variables in this model to incorporate all significant findings from the correlation analysis. This decision, although compromising model stability, was crucial for a comprehensive explanation of our results.

**TABLE 7:** Multiple linear regression model with neuroimaging features explaining migraine frequency, after controlling for age, BMI, and disease duration.

Model	Predictors	β	SE	*T* score	*p*-Value	VIF	*R* ^2^	D-W value
1	Age	−0.487	0.742	−0.656	0.516	1.495	0.105	0.763
	BMI	1.375	1.560	0.882	0.384	1.447		
	Duration	0.342	0.226	1.513	0.140	1.196		
2	Age	−0.078	0.506	−0.155	0.878	1.520	0.617	2.067
	BMI	1.038	1.061	0.978	0.336	1.465		
	Duration	0.114	0.163	0.702	0.488	1.361		
	ROI 6 to C1	1.659	0.404	4.109	<0.001**	1.053		
	ROI 1	−2.587	0.654	−3.953	<0.001**	1.177		
3	Age	−0.496	0.458	−1.084	0.288	1.641	0.738	1.856
	BMI	0.813	0.928	0.876	0.389	1.477		
	Duration	0.115	0.145	0.790	0.436	1.420		
	ROI 6 to C1	1.253	0.389	3.217	0.003**	1.290		
	ROI 6 to C2	−0.505	0.375	−1.347	0.189	2.072		
	ROI 1	−1.571	0.765	−2.054	0.049*	2.118		
	ROI 4	−0.991	0.596	−1.662	0.108	1.644		
	ROI 6	−1.070	0.621	−1.722	0.096	1.409		

*p < 0.05; **p < 0.01; n = 36. β, parameter estimate; SE, standard error; VIF, Variance Inflation Factor; D-W value, Durbin-Watson value; BMI, body mass index; ROI 6 to C1, the rsFC of left SPG to left MOG; ROI 6 to C2, the rsFC of left SPG to left AG; ROI 1, right supramarginal gyrus; ROI 4, left middle occipital gyrus; ROI 6, left superior parietal gyrus.

## Discussion

To our knowledge, no previous studies have simultaneously analyzed the vestibular function and the structural and functional MRI features in CM and EM patients. In the present study, CM patients had more vestibular symptoms, higher DHI scores, and lower saccade accuracy than EM and HC. The functional and structural changes in CM and EM patients were evaluated by VBM, mALFF, and rsFC analysis. VBM found no structural differences among the three groups. Compared with the HC group, altered mALFF values could be observed in the right SMG, left AG, left MFG, left MOG, right Rol, and left SPG in the CM and/or EM group. Additionally, altered rsFC of the left SPG with the left MOG and left AG were observed. Besides, the rsFC of the left SPG with the left MOG was significantly correlated with migraine frequency and vestibular dysfunction features. Therefore, these findings indicated that the vestibular dysfunction is related to altered spontaneous neural activity in brain regions involved with sensory integration and processing. This could provide new insights into the pathological mechanisms of migraine chronification associated with vestibular dysfunction.

### Alterations in vestibular function in migraine

In the current study, 17/18 CM and 13/18 EM patients had vestibular symptoms. They had a total DHI score of 37.29 ± 24.36 in the CM group compared to 18.46 ± 16.68 in the EM group, with the most significant differences derived from physical aspects, in line with previous research. Previous studies have revealed that CM patients have significantly higher DHI scores than EM patients, the DHI for CM is from 31.9 to 51.1 on average, and the EM is 22.1 to 44.3 (Carvalho et al., 2019, 2022; Lee S. et al., 2019). The two groups of migraine patients in our study had relatively low DHI scores compared to the highest scores in other studies. This could be associated with the inclusion of VM patients in their study. The DHI score for VM patients was reported to be around 50 (Sugaya et al., 2017). VM patients were excluded, which could be the main reason for the lower DHI scores in our study. These results depict that the frequency and severity of vestibular dysfunction are higher in CM than in EM patients.

The study found few differences in vestibular function assessment among three groups, except for a significant difference in saccade accuracy. The CM group had lower accuracy, indicating possible differences in the ability to control eye movements between the groups. Saccade accuracy is controlled by the brainstem and cerebellum (Robinson et al., 1993; Takahashi et al., 2022). Therefore, the decrease in accuracy could reflect more severely impaired brainstem or cerebellum function in CM patients. Coppola et al. (2017) observed that GMV of the cerebellar hemispheres in CM patients was negatively associated with migraine duration and positively correlated with the number of medications received per month. A recent proton magnetic resonance spectroscopy study also observed that the dentate nucleus and periaqueductal gray had significantly lower gamma-aminobutyric acid (GABA) levels in CM patients (Wang W. et al., 2022). These results may explain the decline in saccade accuracy within the CM group.

It is important to note that the negative result is not worthless and may imply that the vestibular dysfunction that occurs during the migraine chronification process is more rely on the central mechanisms. More comparisons of the vestibular examination between the CM and EM groups would be beneficial in understanding migraine chronification. In addition, the vestibular analysis in this study was performed during the interictal period, which could be associated with fewer between-group differences. Therefore, future studies could be carried out during the ictal period to obtain more valuable data.

### Altered structural features in patients with migraine

In our study, the VBM analysis did not reveal any significant structural impairment. The presence of GMV alterations in migraine patients has been a topic of discussion in the literature, with studies and meta-analyses yielding heterogeneous results (Wang et al., 2020; Masson et al., 2021; Chen et al., 2022a; Zhang et al., 2023). Some researchers argue that there are no significant changes in brain structure in migraine patients (Wang et al., 2020; Masson et al., 2021), which is consistent with our findings. However, it is important to note that this topic remains an area of ongoing debate and may warrant further investigation to better understand the role of structural changes in migraine pathophysiology.

### Altered functional features of multisensory integration regions in patients with chronic migraine

The majority of the abnormal brain regions identified in this study are located in the parietal lobe, such as the SPG, SMG, and AG, and the Rol is anatomically close to the parietal lobe. The parietal lobe, as the center of sensory processing, is inevitably closely associated with the processing and transmission of nociceptive information, but the specific role of its various sub-regions in this is differentiated.

The SPG, part of the parietofrontal network, could be the pain processing matrix (Garcia-Larrea and Peyron, 2013). Previous neuroimaging studies have identified structural and functional alterations in this brain region among migraine patients (Hu et al., 2019; Wang Z. et al., 2022). The SPG may modulate nociceptive processing by forming functional connections between brain regions involved in nociceptive processing, such as the red nucleus (Huang et al., 2019) and brainstem (Qin et al., 2020). Hence, impaired brain activity in the SPG could have a critical role in the nociceptive information processing.

The SMG is an integral part of the inferior parietal lobe and is primarily involved in the cognitive assessment of pain (Lamm et al., 2011). A previous study identified structural alterations in the SMG associated with functional abnormalities in this brain region in CM patients (Chiapparini et al., 2010). This decrease in neural activity of the SMG has been observed in untreated CM with medication overuse headache (MOH) and could be reversed after treatment (Grazzi et al., 2010; Ferraro et al., 2012). This region also connects with the pulvinar (Huang and Barber, 2021), a thalamus region associated with migraine-related pain and allodynia (Burstein et al., 2010). These findings indicate that the SMG may modify pain sensory discrimination abilities and complex regulatory mechanisms of patients contributing to pain processing. Alterations in this brain region could disrupt the cognitive evaluation of pain.

The AG is close to the temporal and occipital lobe junction. The association between migraine and AG is not yet fully understood. Studies have shown that migraine patients may exhibit abnormal pain perception (Maleki et al., 2012; Schwedt and Chong, 2014). These abnormalities may be related to functional changes in the AG, given its functions in cognitive functions such as pain processing, pain memory, and pain-related emotional responses (Xue et al., 2012; Seghier, 2013).

The MFG in the frontal lobe is responsible for pain modulation and emotion regulation (Briggs et al., 2021). Studies have shown alterations in the MFG, including thinner cortices in CM patients compared to HCs (Magon et al., 2019; Lai et al., 2020). Migraineurs with high-frequency attacks also exhibited increased activity in the left MFG compared to those with low-frequency attacks (Chen et al., 2019). Altered rsFC between the thalamus and the MFG has been observed in VM and EM patients (Cao et al., 2022; Chen et al., 2022b), which also further implies its relevance to sensory processing. The involvement of the prefrontal cortex (including MFG) in emotional and cognitive aspects of pain indicates that its activation in migraine patients could be associated with negative cognition of pain and an inability to cope with it effectively. Further research is needed to fully understand the precise contribution of the MFG to migraines.

The Rol, located on the precentral and postcentral gyri, has been implicated in emotional processing and sensory integration (Sutoko et al., 2020; Triarhou, 2021). A meta-analysis of VBM studies detected GMV abnormalities in the Rol among migraine patients (Zhang et al., 2023). The specific role of the rolandic operculum in migraine, whether it is related to pain or accompanying negative emotions, is still not well understood.

While almost of these brain regions play a role in pain processing, they are also crucial nodes for multisensory processing and integration. The SPG plays a crucial role in vestibular function by processing and integrating vestibular information related to spatial orientation, localization, and balance (Gurvich et al., 2013). It receives vestibular projections from the thalamus and collaborates with other brain regions, such as the superior temporal gyrus and the posterior insula, to integrate visual, vestibular, and somatosensory information (Lopez and Blanke, 2011). This multisensory integration contributes to maintain spatial orientation and postural control, which are essential for coordinating movement and understanding the body’s position in the environment (Guldin and Grüsser, 1998). Structural and functional alterations in the SPG can also be observed in vestibular disorders. Compared to controls, VM patients indicated significantly thinner cerebral cortex thickness in the right SPG (Zhe et al., 2021). One fMRI study on chronic unilateral vestibulopathy (CUVP) revealed that patients had bilaterally reduced ReHo values in the SPG (Si et al., 2022).

The SMG in humans could have evolved from the lateral intraparietal cortex in primates and may be associated with spatial signal processing (Subramanian and Colby, 2014). In conjunction with the superior temporal gyrus and the posterior insula, the SMG region can receive and integrate visual, vestibular, and somatosensory information into the spatial orientation structure (Biesbroek et al., 2014; Drag et al., 2016).

The AG is also an essential part of the default mode network (DMN) and is thought to be the hub for integrating global sensory information (de Pasquale et al., 2012). One previous study observed that the elevated rsFC between the bilateral AG and the dorsolateral prefrontal cortex (dlPFC) in patients was significantly higher, which may explain the fact that MWoA patients have more abnormalities in spatial information discrimination than HCs (Koenigs et al., 2009).

These regions are important components of multisensory integration system, which process multiple senses including nociception, somatosensory, vestibular sensory, and vision. Consequently, impairments to these brain regions may induce multiple sensory deficits. Abnormal function of multisensory cortical regions is one of the current hypotheses regarding the production of vestibular dysfunction in migraine (Lempert and Neuhauser, 2005; Zhang et al., 2020). Our study provides additional evidence for this hypothesis. The present study suggests that migraine chronification may lead to functional alterations in some brain regions that are involved not only in nociceptive processing, but also in multisensory processing and integration including visual, vestibular, and somatosensory information. Therefore, impairment to these brain regions may cause various vestibular dysfunction in patients with CM.

### Resting-state functional connectivity alterations in migraine chronification

Then we performed an ROI-based rsFC analysis. When the SPG was set as ROI, the CM group showed enhanced rsFC between the left SPG and left MOG than the EM and HC groups. The role of the occipital lobe in migraine is thought to be related to visual aura (Bridge et al., 2015; Lai et al., 2016; Niddam et al., 2016). However, recent studies have showed that alterations in the occipital lobe can also be observed in migraines without aura (MWoA) (Li et al., 2020; Wei et al., 2020; Yin et al., 2020). As a center for auditory and visual integration, it may contribute to photophobia and phonophobia onset (Beauchamp, 2005). It is important to note that these two brain regions are not only involved in the development of migraine, but they are also important components of the human vestibular cortex (Fasold et al., 2002; Gurvich et al., 2013). The visual cortex is essential for spatial orientation, which may also be how it maintains balance. Many vestibular disorders are also related to dysfunction in these regions. CUVP patients exhibited significantly decreased mALFF values in the right MOG compared to HCs (Si et al., 2022). Another rs-fMRI study identified reduced rsFC between the vestibular regions of the parietal operculum and the visual association areas of the middle occipital gyrus among participants suffering from Mal de debarquement syndrome (Jeon et al., 2020). This evidence suggests that MOG may be involved in the pathophysiology of vestibular-related disorders through visual mechanisms.

The concept of an inhibitory reciprocal vestibulo-visual interaction contributes to the understanding of our findings. The other sensation will change in the opposite direction when the input to one of the sensory signals changes (Deutschländer et al., 2002). Significantly lower mALFF values in the SPG of migraine patients may indicate a declining function in multisensory integration. Consequently, the brain may increase its reliance on vision, enhancing the rsFC between the SPG and MOG to maintain balance. Correlation analysis demonstrated that rsFC between SPG and MOG enhanced with migraine frequency, suggesting that migraine chronification exacerbates multisensory integration impairment. Several studies support our hypothesis. Studies have found that frequent migraine attacks negatively affected postural control performance using a modified sensory organization test (SOT) and a stability limit test (Carvalho et al., 2013, 2017). The vestibular system indicated lower SOT scores, followed by the visual and somatosensory systems (Carvalho et al., 2022). This may depict the degree and order of impairment of the balance system among migraineurs. Additionally, migraineurs combined with vestibular dysfunction also perform worse in static posturography and rely more on the vision for balance control (Carvalho et al., 2022). Moreover, altered vestibulo-visual cortical interactions are reported in VM patients (Bednarczuk et al., 2019). A recent study found that VM patients exhibited more elevated vision dependence and lower stability of the postural control system when standing (Lim et al., 2018). In another VM study, 71.3% of these patients had visually induced vestibular symptoms, and 82.2% reported position-motion-induced vestibular symptoms (Vuralli et al., 2018). These results suggest that common vestibular symptoms in VM patients could be associated with vestibulo-visual cortical interaction dysfunction. Our study implies that a similar mechanism may underlie vestibular dysfunction in CM patients. The nature of the multisensory integration system impairment caused by migraine chronification may be an abnormality and mismatch in the input ratio of visual, vestibular, and somatosensory signals.

Another finding of the present study is the altered rsFC between the left SPG and left AG among the three groups. One fMRI study utilized independent component analysis and found increased connectivity between the CM and HC groups in the left AG and the right SPG (Zou et al., 2021). This is consistent with our findings to a certain extent. Moreover, SPG and AG are close to each other in the brain and can interact to support various cognitive functions. They are parts of the parietal lobe and involve processes including language, attention, and spatial awareness (Seghier, 2013). The AG and SPG can integrate information from different senses and transform them into a coherent environmental representation (Sack, 2009). The rsFC between AG and SPG was negatively correlated with migraine frequency, indicating that the migraine chronification caused a deterioration in the cooperative sensory integration between the two brain regions.

### Neuroimaging features concurrently correlated to migraine frequency and vestibular parameters

In the correlation analysis, we found that some neuroimaging features correlated with both migraine frequency and vestibular indications. The rsFC between the left SPG and left MOG exhibited a significant positive correlation with both migraine frequency and the DHI total score, while showing a significant negative correlation with saccade accuracy, which may suggest that this altered rsFC may be involved in both the migraine chronification and the development of vestibular dysfunction. Multiple linear regression analysis revealed that the *Z* value of the rsFC between left SPG to left MOG was a significant predictor of both total DHI score and migraine frequency. A higher *Z* value of this rsFC was associated with greater severity of vestibular dysfunction and increased frequency of migraines, even after controlling for age, BMI, and disease duration. Previous studies have demonstrated the correlation between migraine frequency and the parietal lobe or occipital lobe (Lai et al., 2016; Schwedt et al., 2017; Yin et al., 2020). The above studies are consistent with the results we have obtained, and our study further suggests that abnormal FC between the parietal and occipital lobes may be the key region for the development of vestibular dysfunction during migraine chronification.

Another region is the SMG, where the mALFF values demonstrated a significant correlation with both migraine frequency and saccade accuracy. Multiple linear regression further confirmed the predictive role of the SMG in migraine frequency. However, no predictive effect of the SMG on DHI changes was found, possibly indicating a minimal or indirect impact of the SMG region on vestibular function. The latency and accuracy of the saccades are primarily related to the brainstem and cerebellar function (Tanaka et al., 2021; Takahashi et al., 2022), however, the prefrontal area and visual pathways are also involved in the saccade process. The SMG region is capable of receiving and integrating visual, vestibular, and somatosensory signals (Biesbroek et al., 2014; Drag et al., 2016). A previous study indicated that the SMG is sensitive to transsaccadic changes within visual stimuli (Dunkley et al., 2016). In a recent fMRI study, SMG showed enhanced rsFC with both prefrontal saccade regions and anterior intraparietal sulcus/superior parietal lobule (Baltaretu et al., 2020). This may also explain the altered function of the MFG region in CM patients. These results support the involvement of SMG in the saccade function integration. Therefore, the migraine chronification can aggravate the abnormal SMG function, which may impair the saccade processing pathway and ultimately cause reduced accuracy.

These findings reveal key regions involved in the development of migraine chronification and vestibular dysfunction. The results suggest that as migraine frequency increases, functional abnormalities in the multisensory integration system may be the underlying cause of vestibular dysfunction, which is consistent with our hypothesis. More importantly, the abnormal functional features identified in this study could serve as a bridge linking migraine chronification and vestibular dysfunction, which provides crucial insights for further research and therapeutic interventions.

It should be noted that our findings only suggest a correlation between the migraine chronification and neuroimaging characteristics, and are not sufficient to establish a causal relationship. These changes may be bidirectional. It is unclear whether chronification cause these changes or if these changes contribute to the chronicity of migraines. Further longitudinal studies are needed to better understand the bidirectional relationship. By tracking patients’ progression and observing dynamic changes, we can gather evidence to validate the causal relationship. Advanced neuroimaging techniques like task-based fMRI, along with measurements of physiological indicators and neural activity, can also contribute to unravel the complex interactions between these alterations.

### Limitations

The present study has several limitations for consideration. First, the limited number of participants in this study could have reduced the ability to detect meaningful differences or connections between variables. Additionally, migraineurs exhibit various vestibular symptoms, and further differentiation of their types would expose this issue. Another problem is that the vestibular symptoms in migraineurs are not always the same in every episode, leading to difficulty in calculating the frequency of their vestibular dysfunction. Perhaps a subgroup analysis with a larger sample size would help to resolve this dilemma. Secondly, our study found not many abnormalities in the vestibular function tests between the CM and EM groups. This may be because we performed the vestibular examination during the interictal period in the quest for uniformity between the MRI and the vestibular data. More differences could be obtained by examining the ictal period. However, the tolerability of MRI and vestibular examination in migraine patients could become an issue when conducting studies during the ictal period. Thirdly, this study utilized a cross-sectional design, lacking longitudinal or repeated measures assessments. Consequently, our understanding of variables’ temporal changes and developmental trends is limited. The absence of long-term tracking data prevents us from determining the extent and direction of variable changes across different time points, while the single-timepoint observation and measurement impede control over individual differences and potential confounders, potentially introducing bias into the study results. Further longitudinal studies are necessary to verify these hypotheses and causation. In addition, the majority of patients included in this study were female, which may lead to incorrect general conclusions. Further controls for gender may be needed when designing such studies in the future.

## Conclusion

Structural and functional MRI data in the present study has indicated that the functional alterations due to migraine chronification were located in the right SMG, left AG, left MFG, left MOG, right Rol, and left SPG. Most abnormal functional brain regions are associated with multisensory integration involved in processing the vestibular sensory, somatosensory, and visual sensations for maintaining balance. Seed-based FC analyses were performed in these brain regions and indicated aberrant FC between left SPG with MOG and AG. The mALFF values of SMG and the rsFC of SPG with MOG correlated with both headache frequency and indicators of vestibular dysfunction. Of particular significance is the abnormal rsFC between the SPG and MOG, which was confirmed through multiple linear regression. These are the brain regions may responsible for the migraine chronification causing vestibular symptom development. The abnormal rsFC of the SPG with MOG in the CM group may be associated with vestibulo-visual cortical interaction dysfunction. This could be a potential mechanism and therapeutic target for developing vestibular dysfunction in migraine patients. Our study indicated new insights within the pathophysiological mechanisms of migraine chronification and vestibular dysfunction via neuroimaging evidence.

## Data availability statement

The original contributions presented in this study are included in the article/Supplementary material, further inquiries can be directed to the corresponding author.

## Ethics statement

The Ethics Committee of First Affiliated Hospital of Chongqing Medical University approved the study. The patients/participants provided their written informed consent to participate in this study.

## Author contributions

LD and JZ conceived and designed the study. LD, XF, YF, HL, and XL recruited the participants. LD and HL analyzed the data. LD and XF drafted the manuscript. LD, XF, and JZ revised the manuscript. All authors contributed to the article and approved the submitted version.
