# Supplementary material for: Impairments to the multisensory integration brain regions during migraine chronification: correlation with the vestibular dysfunction
Source: Front Mol Neurosci. 2023 Jul 3;16:1153641. doi: 10.3389/fnmol.2023.1153641 (PMC10350528; doi:10.3389/fnmol.2023.1153641)
Supplement: Supplementary file 1 [file Data_Sheet_1.docx]

**Supplementary Table 1. Multiple linear regression model with neuroimaging features explaining migraine frequency, after controlling for age, BMI, and disease duration**

| Model | Predictors | β | SE |  | T score | *p* value | VIF | R^2^ | D-W value |
| --- | --- | --- | --- | --- | --- | --- | --- | --- | --- |
| 1 | Age | 0.081 | 0.177 | 0.093 | 0.459 | 0.650 | 1.581 | 0.173 | 0.692 |
|  | BMI | 0.096 | 0.617 | 0.031 | 0.156 | 0.877 | 1.505 |  |  |
|  | Duration | 0.369 | 0.195 | 0.349 | 1.894 | 0.067 | 1.318 |  |  |
| 2 | Age | 0.200 | 0.134 | 0.229 | 1.490 | 0.147 | 1.628 | 0.566 | 1.683 |
|  | BMI | 0.044 | 0.463 | 0.014 | 0.096 | 0.924 | 1.519 |  |  |
|  | Duration | 0.125 | 0.159 | 0.119 | 0.787 | 0.438 | 1.569 |  |  |
|  | ROI 6 to C1** | 17.065 | 5.188 | 0.406 | 3.289 | 0.003 | 1.054 |  |  |
|  | ROI 1** | -20.283 | 6.033 | -0.449 | -3.362 | 0.002 | 1.236 |  |  |
| 3 | Age | 0.103 | 0.135 | 0.117 | 0.759 | 0.455 | 1.808 | 0.644 | 1.548 |
|  | BMI | -0.039 | 0.444 | -0.013 | -0.089 | 0.930 | 1.530 |  |  |
|  | Duration | 0.143 | 0.158 | 0.135 | 0.906 | 0.373 | 1.686 |  |  |
|  | ROI 6 to C1* | 11.898 | 5.526 | 0.283 | 2.153 | 0.040 | 1.310 |  |  |
|  | ROI 6 to C2 | -5.565 | 4.340 | -0.201 | -1.282 | 0.211 | 1.870 |  |  |
|  | ROI 1 | -13.858 | 7.577 | -0.307 | -1.829 | 0.078 | 2.136 |  |  |
|  | ROI 4 | -5.288 | 6.666 | -0.117 | -0.793 | 0.434 | 1.657 |  |  |
|  | ROI 6 | -11.606 | 8.646 | -0.178 | -1.342 | 0.191 | 1.338 |  |  |

n=36

β, parameter estimate; SE, standard error; VIF, Variance Inflation Factor; D-W value, Durbin-Watson value; BMI, Body Mass Index; ROI 6 to C1, the rsFC of left SPG to left MOG; ROI 6 to C2, the rsFC of left SPG to left AG; ROI 1, right supramarginal gyrus; ROI 4, left middle occipital gyrus; ROI 6, left superior parietal gyrus.


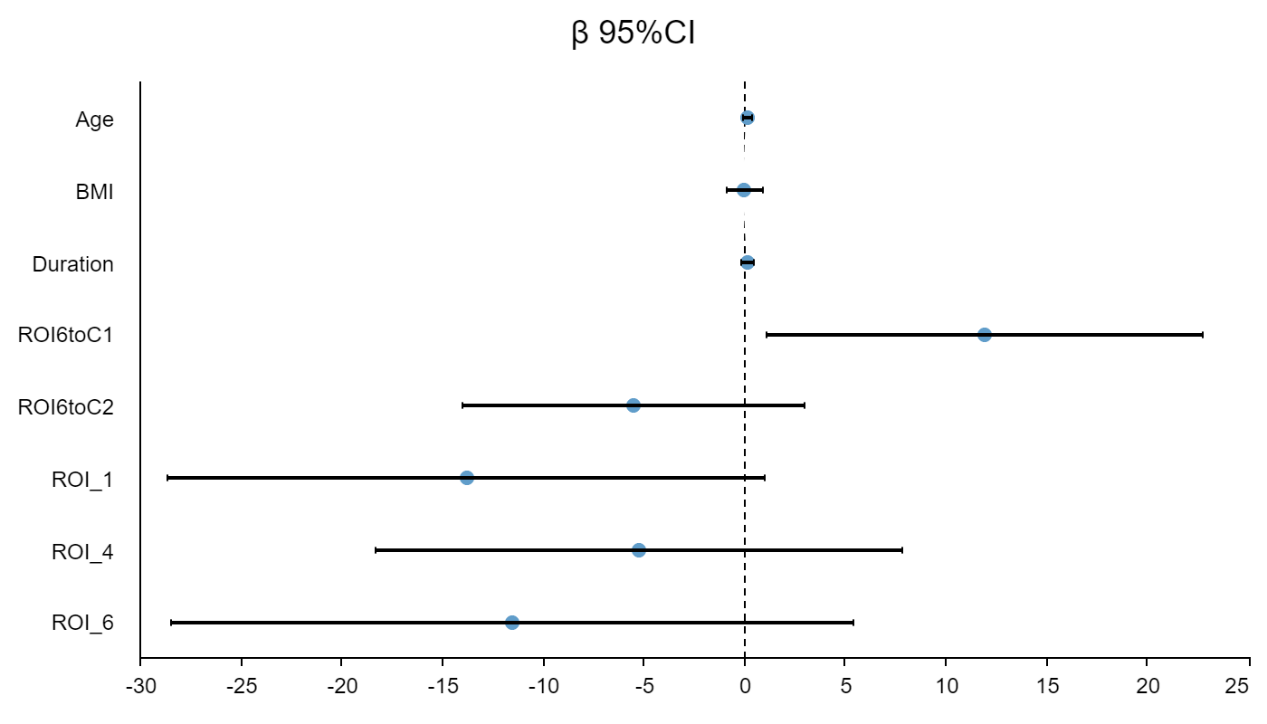


**Supplementary Figure 1. The Regression coefficients in this Multiple linear regression model with neuroimaging features explaining migraine frequency, after controlling for age, BMI, and disease duration**


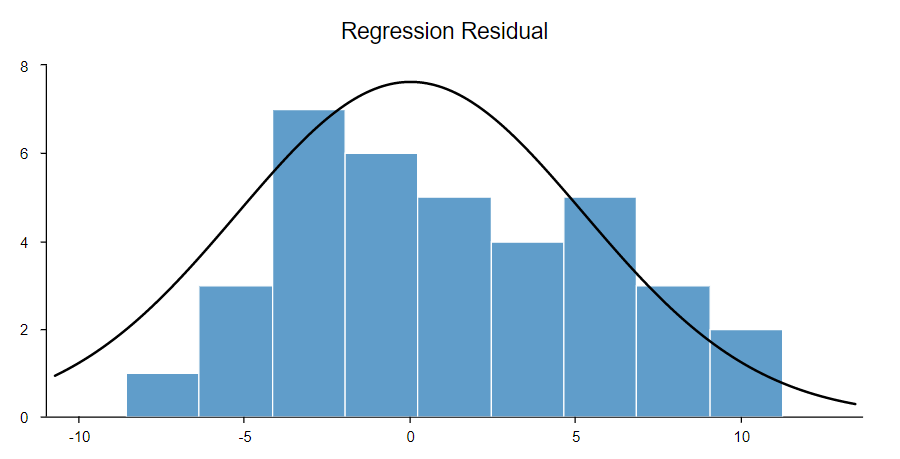


**Supplementary Figure 2. The Histogram of regression residuals in this Multiple linear regression model with neuroimaging features explaining migraine frequency, after controlling for age, BMI, and disease duration**


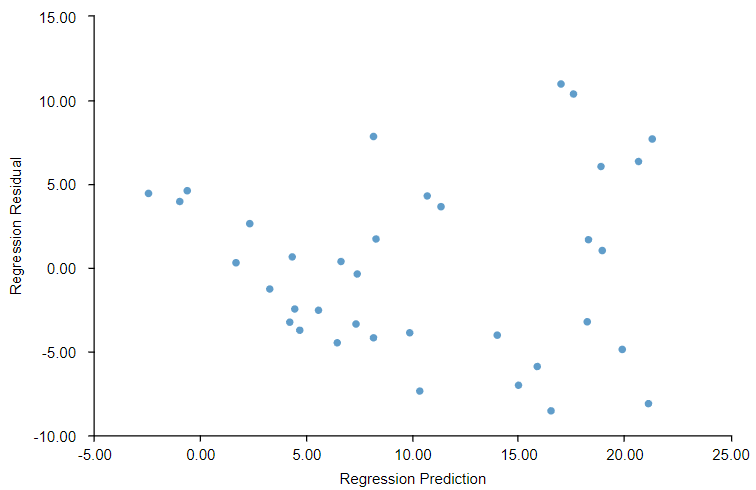


**Supplementary Figure 3. The Scatterplot of regression residuals in this Multiple linear regression model with neuroimaging features explaining migraine frequency, after controlling for age, BMI, and disease duration**

The residual plot analysis method plots the residuals on the y-axis and other appropriate variables on the x-axis. If the points on the residual plot are randomly scattered without any pattern, it is considered to conform to the assumption. However, if the points on the residual plot exhibit a certain trend or show a clear pattern, it is considered to have heteroscedasticity. Based on this, the scatter plot exhibits certain heteroscedasticity. There are a number of ways to deal with heteroskedasticity, and in this article we have chosen to standardize the logarithm of the variables to reduce heteroskedasticity. The model based on the transformed variables can be found in table 7 in the main body of the article. Again, we tested the residuals of the newly created model for normality and heteroskedasticity.


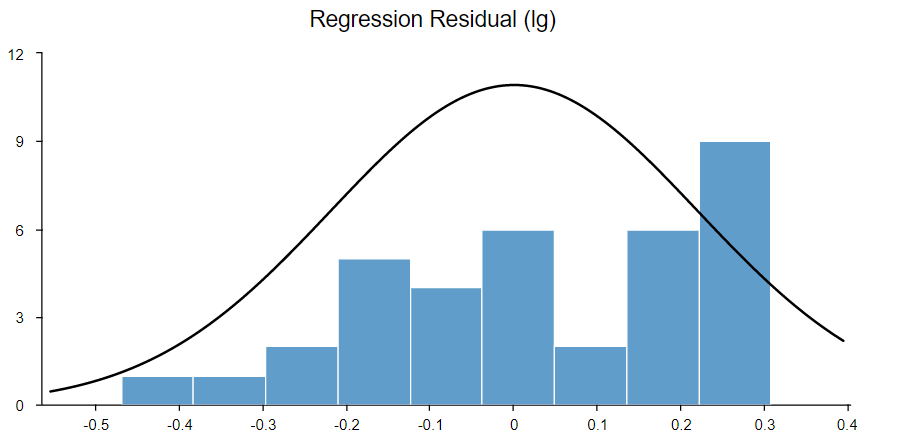


**Supplementary Figure 4. The Histogram of regression residuals in this Multiple linear regression after standardize the logarithm of the variables**


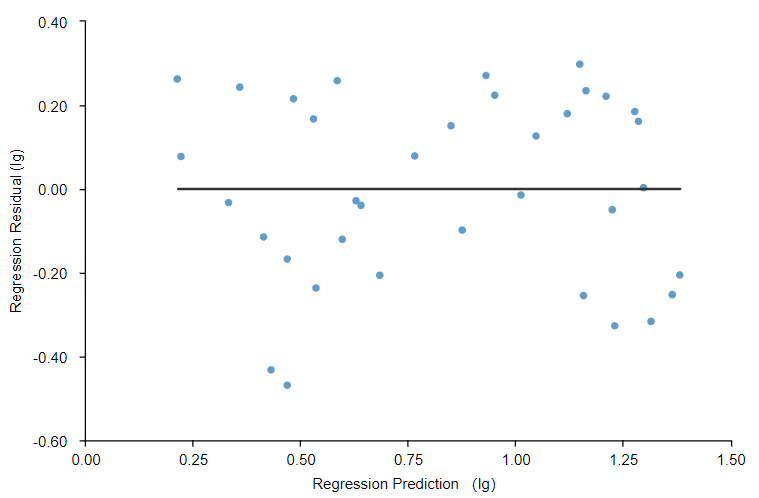


**Supplementary Figure 5. The Scatterplot of regression residuals in this Multiple linear regression after standardize the logarithm of the variables**

As can be seen, there is a partial loss of normality, but it is still within acceptable limits. Importantly, the heteroskedasticity has been improved to a large extent.
